# Anterior chamber depth and vitreous zonule integrity as biomarkers for intraoperative capsule laxity in angle closure

**DOI:** 10.1016/j.aopr.2026.04.002

**Published:** 2026-05-01

**Authors:** Hui Zhang, Jin Wang, Yue Wang, Zijian Liu, Dongjun Li, Lin Shen, Chunyan Qiao, Ching-Yu Cheng, Ye Zhang

**Affiliations:** a Beijing Tongren Eye Center, Beijing Key Laboratory of Ophthalmology and Visual Science, Beijing Tongren Hospital, Capital Medical University, Beijing, China; b Beijing Institute of Ophthalmology, Beijing, China; c Centre for Innovation and Precision Eye Health, Yong Loo Lin School of Medicine, National University of Singapore, Singapore; d Department of Ophthalmology, Yong Loo Lin School of Medicine, National University of Singapore, Singapore; e Singapore Eye Research Institute, Singapore National Eye Centre, Singapore

**Keywords:** Capsule laxity, Angle closure, Preoperative biomarkers, Vitreous zonule

## Abstract

**Objective:**

To investigate preoperative predictors for anterior and posterior capsule laxity during phacoemulsification combined with anti-glaucoma surgery in eyes with angle closure.

**Methods:**

Cross-sectional study. Patients aged ≥40 years diagnosed with angle closure who underwent phacoemulsification combined with anti-glaucoma surgery between May 2023 and May 2025 were enrolled. Eyes were classified into four groups based on intraoperative findings: anterior capsule laxity, posterior capsule laxity, combined capsule laxity, and normal capsule. Mixed-effects logistic regression analysis was employed to determine the independent predictors of anterior and posterior capsule laxity, respectively.

**Results:**

A total of 241 eyes of 203 patients were included. 36 eyes (14.9%) had anterior laxity, 51 eyes (21.2%) had posterior laxity, 73 eyes (30.3%) had combined capsule laxity, and 81 eyes (33.6%) had normal capsule. Eyes with anterior laxity had shallower anterior chamber depth (ACD) than normal capsule eyes (2.05 (1.82,2.29) mm vs. 2.33 (2.13, 2.52) mm, *P*<0.001) and were more frequently acute primary angle closure (APAC) (*P*=0.001). Posterior/combined laxity eyes showed higher vitreous zonule (VZ) absence (both *P*<0.001); combined laxity having thicker lens than normal eyes (5.16 ± 0.35mm vs. 4.95 ± 0.43mm, *P*=0.006). Regression analysis identified APAC diagnosis (odds ratio (OR) 3.04, 95% confidence interval (CI): 1.01–9.38, *P*=0.049) and shallower ACD (OR 0.05, 95% CI: 0.01–0.26, *P*=0.001) as independent predictors for anterior capsule laxity, while APAC diagnosis (OR 3.89, 95% CI: 1.20–13.50, *P*=0.026), VZ absence (OR 0.11, 95% CI: 0.04–0.27, *P*<0.001), and increased ciliary body maximum thickness (CBMT) (OR 1.92, 95% CI: 1.22–3.17, *P*=0.007) predicted posterior capsule laxity. The anterior and posterior capsule laxity prediction models achieved AUC values of 0.817 (95% CI: 0.739–0.896) and 0.781 (95% CI: 0.690–0.872), respectively.

**Conclusions:**

APAC diagnosis and shallow ACD predict anterior capsule laxity, whilst VZ absence, APAC diagnosis, and increased CBMT indicate posterior capsule laxity in eyes with angle closure. Identification of these biomarkers before surgery may help surgeons anticipate complications and optimize postoperative outcomes.

## 1 Introduction

Primary angle closure glaucoma (PACG), as a main type of glaucoma, affected approximately 21.0 million people worldwide in 2020, with 48% of global cases in China (10.1 million).^1^^,^^2^ Blindness caused by PACG accounts for roughly half of global glaucoma-related blindness, and thus PACG imposes a substantial social and economic burden.^3^^,^^4^

In the recent decade, with the evolution of theories on disease mechanisms and surgical techniques, phacoemulsification with intraocular lens implantation (PEI) combined with or without anti-glaucoma procedure has become a first-line surgical treatment for angle closure.^5^, ^6^, ^7^ With the growing number of these procedures, it has become evident that zonulopathy is frequently detected during surgery in patients with angle closure.^8^, ^9^, ^10^, ^11^, ^12^, ^13^ In our previous study, we found that 46.2% of individuals with angle closure exhibited zonulopathy during surgery.^12^

Zonulopathy was identified mainly by the pronounced sign of wrinkling of the anterior capsule with striae formation during continuous curvilinear capsulorhexis (CCC), which represents the anterior capsule laxity of the lens.^8^, ^9^, ^10^, ^11^, ^12^^,^^14^ In our surgical practice, we have noted that posterior capsule laxity is also highly prevalent in patients with angle closure, which manifests as posterior capsule flutter during irrigation/aspiration. Some patients even exhibit combined anterior and posterior capsule laxity. To our knowledge, little attention has been paid to posterior capsule laxity in angle closure so far.

If both anterior and posterior capsule laxity could be anticipated preoperatively, surgeons could tailor parameters for phacoemulsification and irrigation/aspiration, adjust surgical maneuvers, and deploy support devices proactively. Such measures would improve intraoperative safety, minimize complications, and potentially enhance postoperative visual outcomes.

Therefore, this study aimed to investigate differences in ocular characteristics among angle closure patients with different capsular status and to explore potential preoperative predictors for anterior and posterior capsule laxity.

## 2 Materials and methods

### 2.1 Study population

This is an observational, cross-sectional, hospital-based study. Patients aged ≥40 years who were diagnosed with angle closure (including primary angle closure disease (PACD) and acute primary angle closure (APAC)) were consecutively enrolled from Beijing Tongren Hospital, between May 2023 and May 2025. All patients underwent PEI combined with anti-glaucoma surgery (goniosynechialysis with or without goniotomy).

The exclusion criteria were as follows: presentation of secondary angle closure, history of intraocular surgeries (except for laser or surgical peripheral iridotomy), history of ocular trauma, presentation of other ophthalmic diseases that can cause zonulopathy, such as Marfan syndrome, spherophakia, uveitis, retinitis pigmentosa, etc.

Diagnosis of PACD, including primary angle closure (PAC) and PACG, were made based on the criteria proposed by the International Society of Geographical and Epidemiological Ophthalmology.^15^ PAC was defined as an occludable angle (the pigmented posterior trabecular meshwork was not visible at least 180° on gonioscopy) plus peripheral anterior synechiae and/or IOP >21 mmHg but without glaucomatous optic neuropathy (GON). PACG was defined as PAC combined with evidence of GON and visual field defects.

APAC was diagnosed according to the following criteria: (1) the presence of two or more of the following symptoms: eye pain, nausea/vomiting, or an antecedent history of intermittent blurring of vision with halos; (2) presenting marked increased IOP of *>*21 mmHg; (3) the presence of at least three of the following signs: conjunctival injection, corneal edema, a mid-dilated unreactive pupil in affected eyes, or a shallow anterior chamber in both eyes; and (4) gonioscopy or ultrasound biomicroscopic confirmation of an occluded angle in the affected eye.^16^

This study adhered to the tenets of the Declaration of Helsinki regarding research involving human participants. Written informed consent was obtained from all the participants, and the study was approved by the Ethics Committee of the Beijing Tongren Hospital (TREC2022-KY003).

### 2.2 Study examinations

All patients underwent a comprehensive and standardized ophthalmologic evaluation comprising presenting visual acuity (PVA) measurement using logMAR 4-m charts, slit-lamp examination, intraocular pressure (IOP) measurement using a Canon TX-20 tonometer (Canon, Tokyo, Japan), gonioscopic examination using a one-mirror Goldmann lens (Ocular Instruments, Bellevue, WA, USA), optical biometry (including average keratometry (AK) and measurements of central corneal thickness (CCT), central anterior chamber depth (ACD), lens thickness (LT), and axial length (AL)) using IOLMaster 700 (V.5, Carl Zeiss Meditec, Dublin, CA, USA), ultrasound biomicroscopy (UBM) (50 MHz, AVISO, Quantel-medical, France or 50 MHz, MD-300L, Tianjin Maida Medical Technology Co., Ltd., Tianjin, China) and fundus examination using a 90-D lens with a vertical cup-to-disc ratio (VCDR) being assessed and recorded. Patients who used miotics were asked to discontinue use at least three days prior to ocular examinations. Additionally, family history of angle closure, and medical histories of diabetes, hypertension and hyperlipidemia were obtained and recorded through standardized patient interviews.

Gonioscopy was performed in the dark at ×25 magnification by one of two observers (YZ and HZ), who achieved a Cohen's kappa coefficient (*κ*) of 0.83 for the identification of the occludable angle in 20 eyes. Static gonioscopic evaluation was conducted under dim ambient illumination with a shortened slit that did not fall on the pupil. Indentation gonioscopy was subsequently performed with increased illumination to determine the presence of peripheral anterior synechia.

### 2.3 UBM image acquisition and analysis

All patients underwent the UBM examination in a supine position in a lit room. The imaging of both eyes was obtained in the superior, inferior, temporal, and nasal quadrants by two experienced examiners (LS and DL) based on the same standard operation protocol.

UBM parameters were analyzed quantitatively in all four quadrants using ImageJ software (National Institutes of Health, Bethesda, MD) by the same investigator (YW), who was masked to all clinical information. The scleral spur (SS) was identified as the innermost point of the scleral prominence at the junction between the trabecular meshwork and the ciliary body, where the curvature of the inner scleral wall changes from concave (posteriorly) to convex (anteriorly).^17^

**Fig. 1:**
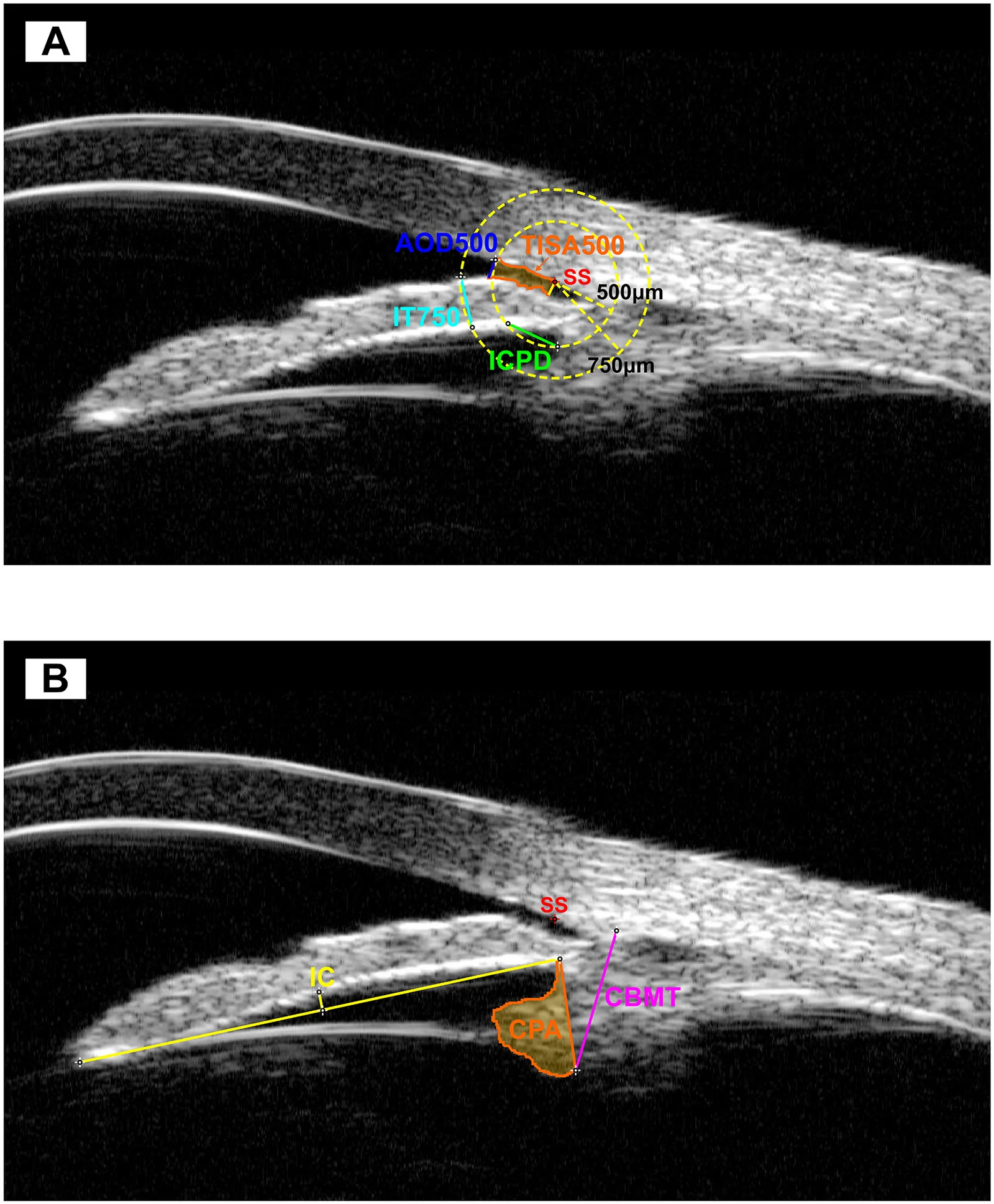
**Anterior Segment Parameters Measured on an Ultrasound Biomicroscopy Image.** Representative ultrasound biomicroscopy images illustrating anterior segment parameter measurements. (A) Measurements of angle-related parameters, including angle opening distance at 500 μm (AOD500), trabecular–iris space area at 500 μm (TISA500), iris thickness at 750 μm from the SS (IT750), and iris–ciliary process distance (ICPD). (B) Measurements of ciliary body-related parameters, including the iris contact point (IC), ciliary body maximum thickness (CBMT), and ciliary process area (CPA). SS, scleral spur; AOD500, angle opening distance at 500 μm; TISA500, trabecular–iris space area at 500 μm; IT750, iris thickness at 750 μm from the SS; ICPD, iris–ciliary process distance; IC, iris contact point; CBMT, ciliary body maximum thickness; CPA, ciliary process area.

The mean values of the parameters from four quadrants were calculated as the final results of each eye, including: (1) angle opening distance at 500 μm (AOD500): the distance between the posterior corneal surface and the anterior iris surface on a line perpendicular to the trabecular meshwork, 500 μm from the SS; (2) trabecular–iris space area at 500 μm (TISA500): the surface area bounded by AOD 500 anteriorly, a line drawn from the SS perpendicular to the plane of the inner scleral wall to the iris posteriorly, the inner corneoscleral wall superiorly, and the iris surface inferiorly; (3) iris thickness at 750 μm from the SS (IT750): the distance from the intersection points on the anterior and posterior surfaces of the iris with a circle of a 750 μm radius centered on the SS; (4) iris curvature (IC): the distance of a perpendicular line extending from a line drawn from the most peripheral to the most central points of the posterior iris to the iris pigment epithelium at the point of greatest convexity; (5) ciliary body maximum thickness (CBMT): the distance from the most inner point of the ciliary processes to the inner wall of sclera or its extended line; (6) iris-ciliary process distance (ICPD): the distance between the iris and the ciliary process intersections with a circle of a 500 μm radius centered on the SS; (7) ciliary process area (CPA): the cross-sectional area of ciliary process bounded by a line connecting the insertion location of iris into the ciliary body and the cross-point of a line at 500 μm from the SS perpendicular to the plane of the inner scleral wall to the ciliary process, and internally by the ciliary process surface (Fig. 1).^17^, ^18^, ^19^, ^20^, ^21^, ^22^, ^23^, ^24^

The intraclass correlation coefficients for all UBM parameters measured by this investigator (YW) ranged from 0.872 to 0.996.

**Fig. 2:**
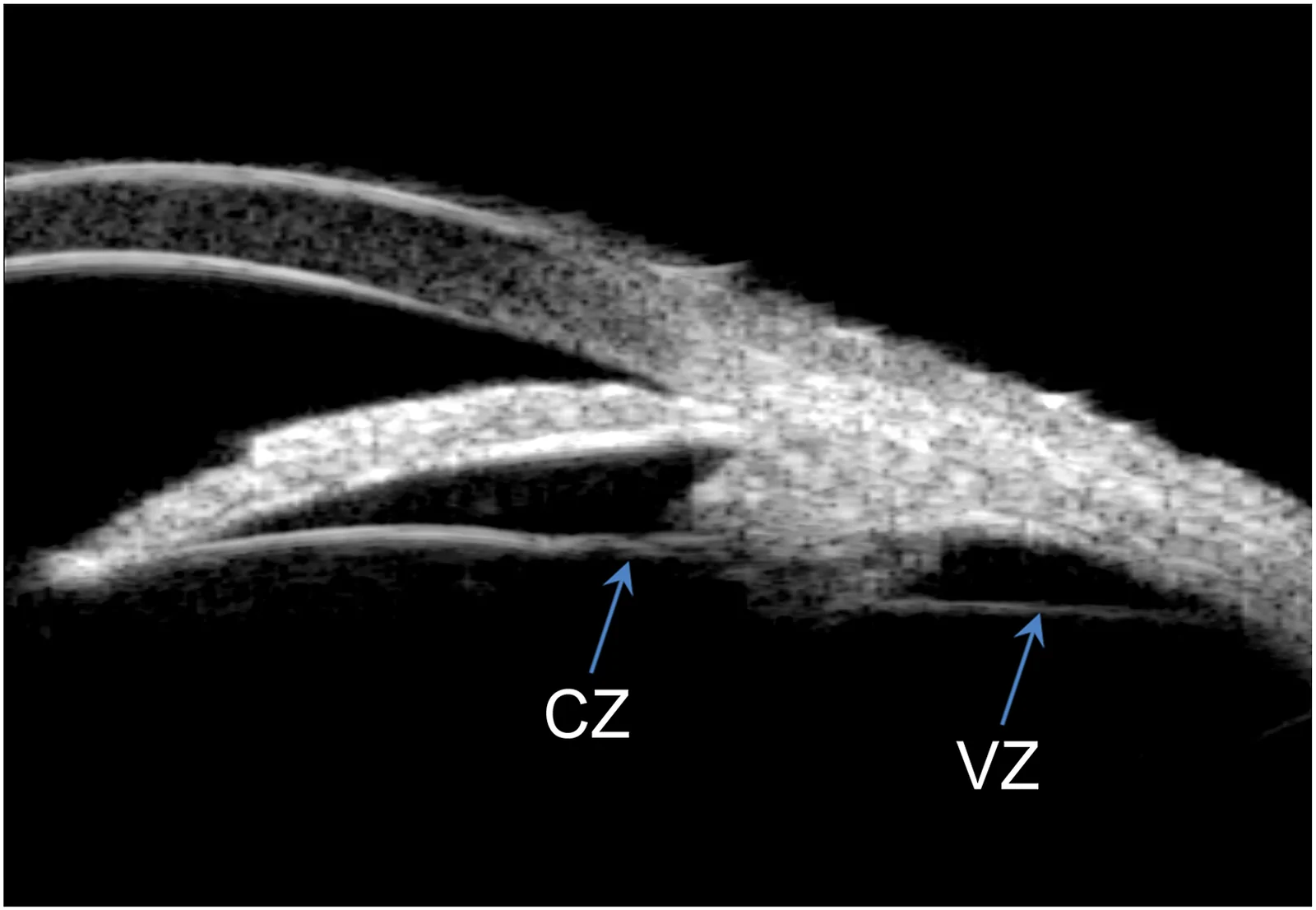
**Ciliary Zonule and Vitreous Zonule on an Ultrasound Biomicroscopy Image.** CZ, ciliary zonule; VZ, vitreous zonule.

The ciliary zonule (CZ) and vitreous zonule (VZ) were assessed on quadrant UBM images. For each eye we recorded (1) zonule presence (defined as visible on ≥1 of the four quadrant images) and (2) the number of quadrants (0–4) showing visible zonular strands (Fig. 2). Zonular visibility was judged by the presence of linear, strand-like echogenic structures in the expected anatomic plane for CZ or in the retrozonular space for VZ. All readings were performed by a single experienced, masked investigator (JW). Eyes with no visible linear zonule-like structures posterior to the ciliary body on any of the four quadrant images were classified as VZ-absence.

### 2.4 Capsule laxity definition and categorization

All the PEI combined with anti-glaucoma surgeries were performed by two experienced glaucoma specialists (HZ and YZ), following a standardized protocol under general or topical anesthesia, with one being the primary surgeon and the other being the first assistant, alternating roles between cases. All procedures were performed using the same phacoemulsification system (Centurion Vision System, Alcon Laboratories, Fort Worth, TX, USA) with standardized fluidic parameters. Specifically, during irrigation/aspiration we used a target IOP of 55 mmHg, a maximum vacuum of 600 mmHg, and an aspiration flow rate of 40 cc/min. These uniform settings were applied in all cases to ensure that observation of posterior-capsule flutter (posterior capsule laxity) was not confounded by variable fluidic conditions.

The determination of capsular status was made intraoperatively by both the primary surgeon and the first assistant independently and documented. Videos of the surgeries were recorded for review. In case of disagreement, a senior experienced glaucoma specialist (CQ) would review the surgical video(s) to make the final diagnosis.

**Fig. 3:**
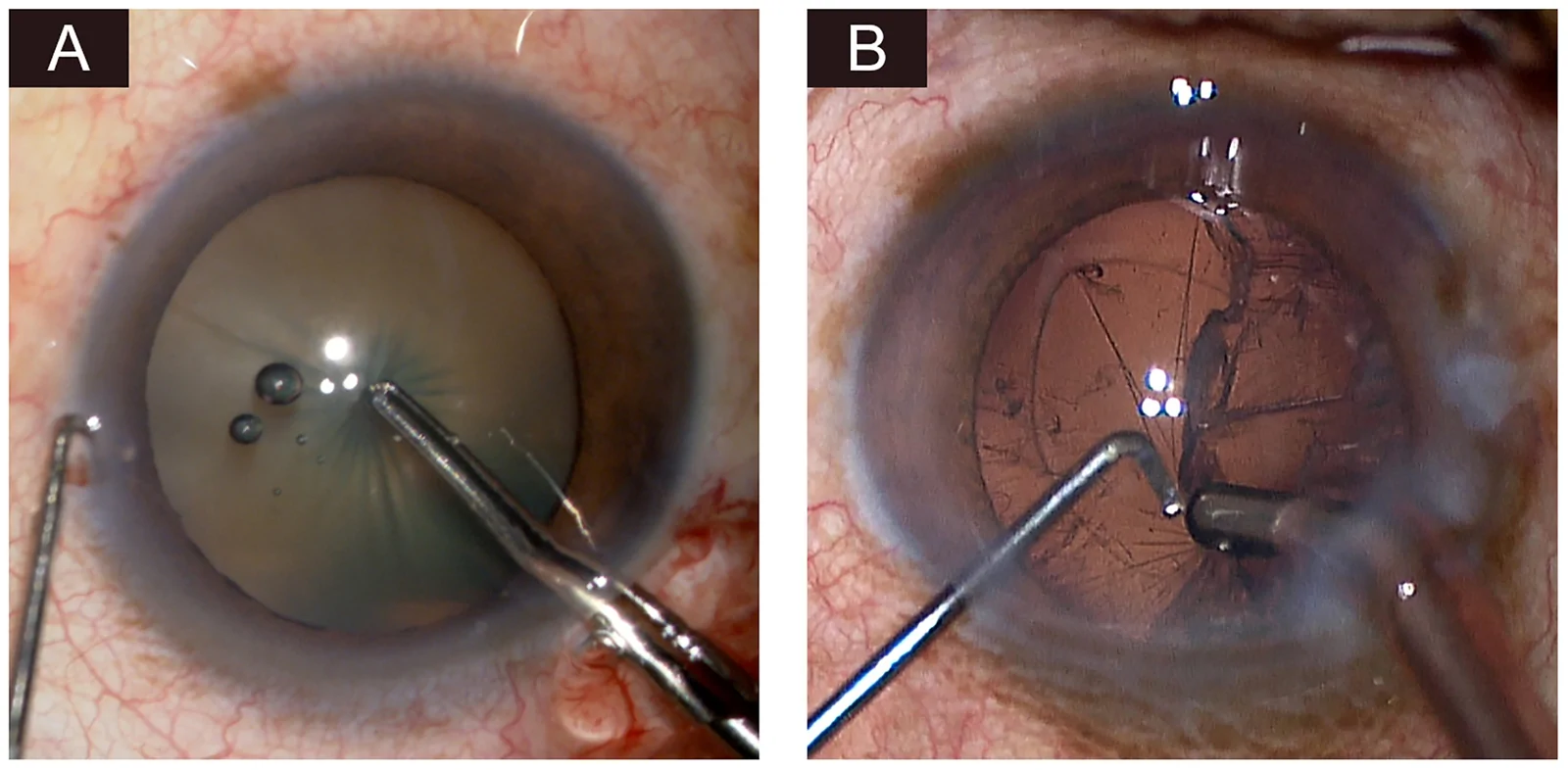
**Intraoperative Signs for Identification of Capsule Laxity.** (A) Wrinkling of lens anterior capsules during capsulorhexis. (B) Floppy posterior capsule with striae formation on the posterior capsule surface during cortex removal.

The capsule status was defined and categorized into four groups based on intraoperative findings (Fig. 3). Anterior capsule laxity was identified by the presence of wrinkling and striae formation on the anterior capsule during capsulorrhexis initiation, with or without difficulty in performing CCC. Posterior capsule laxity was defined as loose or floppy posterior capsule with visible striae formation on the posterior capsule surface during cortical irrigation/aspiration, with or without difficulty in cortex removal. The presence of both signs described above were considered diagnostic of combined capsule laxity and normal capsule was defined as absence of any of the signs indicating capsule laxity.

### 2.5 Statistical analysis

Data from one or both eligible eyes were included for analysis. The normality of continuous data was evaluated using the one-sample Kolmogorov-Smirnov test. Normally distributed continuous data are presented as means ± standard deviation (SD), while non-normally distributed continuous data as medians and interquartile ranges (IQR). Categorical data are presented as frequencies and percentages. One-way ANOVA or Kruskal-Wallis tests for continuous variables and *χ*^2^ test for categorical variables were used to compare differences across the four groups. For normally distributed data, pairwise post-hoc comparisons between groups were performed using Bonferroni-corrected *t*-tests. For non-normally distributed data, pairwise comparisons were performed using Mann-Whitney *U* tests, with a Bonferroni-adjusted significance threshold (*P*<0.05/6 = 0.008) applied to correct for multiple comparisons.

Separate mixed-effects logistic regression models were established to identify the predictors of anterior and posterior capsule laxity, with patient identifiers specified as random effects to account for the potential correlation between eyes from the same individual. In each analysis, eyes with anterior or posterior capsule laxity were compared with those in the normal capsule group. The following variables were included: age, sex, family history of angle closure, diagnosis (APAC vs. PACD), presence of VZ, and ocular biometric parameters measured by IOLMaster and UBM. Continuous variables were standardized using Z-score transformation prior to analysis to analysis to enable comparison of effect sizes across variables measured on different scales and to improve model convergence. Variables with *P*<0.10 in the univariable analysis, based on pairwise post hoc comparisons between each laxity group and the normal capsule group, were included in the multivariable model, which was refined using stepwise selection based on the Akaike Information Criterion. Multicollinearity was assessed using variance inflation factors. Model performance was assessed using receiver operating characteristic (ROC) curve analysis, with area under the curve (AUC) calculated separately for each capsule laxity prediction model.

Variables exhibiting complete separation (i.e., insufficient variability to allow model convergence) were excluded from regression analyses. Specifically, because CZ presence was essentially universal in the normal-capsule group (81/81 eyes), the binary 'Presence of CZ' variable was excluded; the ordinal 'Number of quadrants of CZ' variable was retained for univariable screening.

Statistical analyses were performed using SPSS statistical software version 25.0 (SPSS, Inc., Chicago, IL, USA) and R software (version 4.5.0; R Foundation for Statistical Computing, Vienna, Austria). Statistical significance was set at *P*<0.05.

## 3 Results

### 3.1 Participants and ocular characteristics

A total of 241 eyes of 203 patients with angle closure aged 68.0 (62.0, 72.0) years old were included in the study. 65 (32.0%) were male and 138 (68.0%) were female. Of the included eyes, 36 eyes (14.9%) were in the anterior laxity group, 51 eyes (21.2%) were in the posterior laxity group, 73 eyes (30.3%) were in the combined capsule laxity group, and 81 eyes (33.6%) were in the normal capsule group. Demographic characteristics, including age, sex distribution, systemic comorbidities (hypertension, diabetes, and hyperlipidemia), and family history of angle closure are summarized in Supplementary Table 1.

Ocular characteristics across the four groups are shown Table 1. Significant differences were observed in diagnosis (PACD or APAC) (*P*=0.009), presence of VZ (*P*<0.001), number of quadrants of CZ and VZ on UBM image (both *P*<0.001), VCDR (*P*=0.049), ACD (*P*<0.001) and LT (*P*=0.009) among the four groups. There was no significant difference in PVA, IOP, CCT, AK, AL and presence of CZ (all *P*>0.05).

**Table 1:** Ocular characteristics of the included angle closure eyes with different capsular conditions.

Parameter	1=Anterior capsule laxity group (*n*=36)	2=Posterior capsule laxity group (*n*=51)	3=Combined capsule laxity group (n=73)	4=Normal capsule group (n=81)	*P* value	*P* value (1 & 4)	*P* value (2 & 4)	*P* value (3 & 4)	*P* value (1 & 2)
Diagnosis type, No. (%)									
PACD	23 (63.9)	39 (76.5)	56 (76.7)	73 (90.1)	0.009^d^	0.001^e^	0.033^e^	0.024^e^	0.202^e^
APAC	13 (36.1)	12 (23.5)	17 (23.3)	8 (9.9)					
Type of surgery, No. (%)									
P + I + GSL	25 (69.4)	19 (37.3)	44 (60.3)	33 (40.7)	0.002^d^	0.004^e^	0.690^e^	0.015^e^	0.003^e^
P + I + GSL + KDB	11 (30.6)	32 (62.7)	29 (39.7)	48 (59.3)					
Presence of CZ, No. (%)									
No	3 (8.3)	1 (2.0)	5 (6.8)	0 (0.0)	0.053^d^				
Yes	33 (91.7)	50 (98.0)	68 (93.2)	81 (100.0)					
Number of quadrants of CZ (IQR)	3.0 (2.0, 4.0)	3.0 (3.0, 4.0)	3.0 (2.0, 3.0)	4.0 (3.0, 4.0)	<0.001^a^	0.017^b^	0.139^b^	<0.001^b^	0.222^b^
Presence of VZ, No. (%)									
No	9 (25.0)	27 (52.9)	50 (68.5)	16 (19.8)	<0.001^d^	0.523^e^	<0.001^e^	<0.001^e^	0.009^e^
Yes	27 (75.0)	24 (47.1)	23 (31.5)	65 (80.2)					
Number of quadrants of VZ (IQR)	1.0 (0.3, 2.0)	0.0 (0.0, 2.0)	0.0 (0.0, 1.0)	1.0 (1.0, 2.0)	<0.001^a^	0.521^b^	0.005^b^	<0.001^b^	0.061^b^
PVA (IQR)	0.52 (0.30, 0.70)	0.40 (0.22, 0.70)	0.52 (0.26, 1.30)	0.40 (0.22, 1.05)	0.308^a^				
IOP (IQR), mmHg	15.6 (13.0, 20.8)	17.0 (12.0, 21.8)	17.0 (13.6, 21.0)	17.0 (13.5, 23.1)	0.827^a^				
VCDR (IQR)	0.50 (0.33, 0.78)	0.70 (0.40, 0.80)	0.50 (0.30, 0.80)	0.70 (0.40, 0.90)	0.049^a^	0.098^b^	0.554^b^	0.070^b^	0.031^b^
CCT (IQR), μm	526.0 (495.8, 539.3)	524.5 (495.8, 556.8)	511.5 (486.3, 542.3)	530.0 (507.0, 552.0)	0.103^a^				
AK (IQR), mm	44.61 (43.38, 45.70)	44.07 (42.92, 45.64)	44.67 (43.57, 45.73)	44.72 (43.60, 45.60)	0.757^a^				
ACD (IQR), mm	2.05 (1.82, 2.29)	2.26 (2.03, 2.43)	2.06 (1.85, 2.27)	2.33 (2.13, 2.52)	<0.001^a^	<0.001^b^	0.187^b^	<0.001^b^	0.003^b^
LT (SD), mm	5.06 ± 0.29	5.10 ± 0.39	5.16 ± 0.35	4.95 ± 0.43	0.009^c^	1.000^f^	0.207^f^	0.006^f^	1.000^f^
AL (IQR), mm	22.42 (22.00, 23.03)	22.50 (21.96, 23.20)	22.68 (22.01, 23.29)	22.53 (21.95, 22.89)	0.303^a^				

PACD, primary angle closure disease; APAC, acute primary angle closure; CZ, ciliary zonule; VZ, vitreous zonule; PVA, presenting visual acuity; IOP, intraocular pressure; VCDR, vertical cup disc ratio; CCT, central corneal thickness; AK, average keratometry; ACD, anterior chamber depth; LT, lens thickness; AL, axial length; IQR, interquartile range; SD, standard deviation.

a Kruskal-Wallis test.

b Mann–Whitney test (<0.05/6 = 0.008 = significant difference).

c The one-way ANOVA.

d *χ*^2^ test.

e *χ*^2^ test (<0.05/6 = 0.008 = significant difference).

f Bonferroni test.

Eyes with anterior capsule laxity tended to be APAC (*P*=0.001) compared to the normal capsule group, and had shallower ACD, in comparison with posterior capsule laxity and normal capsule groups (*P*=0.003 and *P*<0.001). Eyes with posterior or combined capsule laxity more frequently showed VZ absence (both *P*<0.001) and fewer quadrants of VZ presence on UBM images (*P*=0.005 and *P*<0.001), compared to eyes with normal capsule. Moreover, eyes with combined capsule laxity had shallower ACD (*P*<0.001), increased LT (*P*=0.006) and fewer quadrants with visible CZ (*P*<0.001) than those with normal capsules.

### 3.2 Anterior segment parameters comparison

A summary of UBM parameters and related comparisons among the four groups are presented in Table 2. No significant differences were found in AOD500, TISA500, IT750, IC, and CBMT (all *P*>0.05), while ICPD (*P*=0.007) and CPA (*P*=0.005) showed significant differences among the four groups. Eyes with combined capsule laxity had larger ICPD (*P*=0.005) and smaller CPA (*P*=0.004) than those with normal capsule.

**Table 2:** UBM parameters in included angle closure eyes with different capsular conditions.

Parameter	1=Anterior capsule laxity group (*n*=36)	2=Posterior capsule laxity group (*n*=51)	3=Combined capsule laxity group (n=73)	4=Normal capsule group (n=81)	*P* value	*P* value (1 & 4)	*P* value (2 & 4)	*P* value (3 & 4)	*P* value (1 & 2)
AOD500 (IQR), mm	0.070 (0.035, 0.156)	0.104 (0.050, 0.186)	0.139 (0.045, 0.217)	0.119 (0.062, 0.214)	0.098^a^				
TISA500 (IQR), mm^2^	0.027 (0.017, 0.053)	0.035 (0.019, 0.061)	0.046 (0.020, 0.070)	0.035 (0.023, 0.063)	0.276^a^				
IT750 (SD), mm	0.376 ± 0.068	0.371 ± 0.082	0.354 ± 0.078	0.391 (0.341, 0.430)	0.074^c^				
IC (IQR), mm	0.158 (0.037, 0.260)	0.148 (0.054, 0.218)	0.133 (0.045, 0.250)	0.078 (0.033, 0.215)	0.249^a^				
CBMT (SD), mm	1.121 ± 0.079	1.156 ± 0.104	1.121 ± 0.121	1.116 ± 0.092	0.187^c^				
ICPD (SD), mm	0.300 ± 0.164	0.261 ± 0.187	0.318 ± 0.180	0.223 ± 0.129	0.007^c^	0.189^d^	1.000^d^	0.005^d^	1.000^d^
CPA (IQR), mm^2^	0.272 (0.221, 0.348)	0.349 (0.273, 0.433)	0.288 (0.203, 0.376)	0.352 (0.270, 0.446)	0.005^a^	0.009^b^	0.773^b^	0.004^b^	0.034^b^

UBM, ultrasound biomicroscopy; AOD500, angle opening distance at 500 μm; TISA500, trabecular-iris space area at 500 μm; IT750, iris thickness at 750 μm from the iris insertion; IC, iris curvature; CBMT, ciliary body maximum thickness; ICPD, iris-ciliary process distance; CPA, ciliary process area; IQR, interquartile range; SD, standard deviation.

a Kruskal-Wallis test.

b Mann–Whitney test (<0.05/6 = 0.008 = significant difference).

c The one-way ANOVA.

d Bonferroni test.

In a subset of 63 eyes that underwent anterior segment optical coherence tomography (AS-OCT), lens vault (LV) differed significantly across the four capsular groups (*P*=0.002; Supplementary Table 2). Post-hoc comparisons revealed that eyes with anterior capsule laxity (1.28 ± 0.38 mm) and combined capsule laxity (1.20 ± 0.32 mm) had significantly greater LV than eyes with normal capsule (0.88 ± 0.34 mm) (*P*=0.002 and *P*=0.010, respectively). To assess representativeness, baseline characteristics of the AS-OCT subset were compared with the remaining eyes; no significant differences were found in age, sex, diagnosis type, ACD, or LT (all *P*>0.05), indicating that this subset is representative of the total cohort.

### 3.3 Predictors for anterior and posterior capsule laxity

Results of univariable and multivariable mixed-effects logistic regression analyses of potential predictors for anterior capsule laxity and posterior capsule laxity are shown in Table 3, Table 4, respectively. For anterior capsule laxity, the univariable logistic regression model identified diagnosis (APAC vs. PACD) (*P*=0.001), ACD (*P*=0.003), AOD500 (*P*=0.037), IC (*P*=0.099), ICPD (*P*=0.016), and CPA (*P*=0.054) as significant potential predictors. In the multivariable model, only the diagnosis of APAC (odds ratio (OR), 3.04; 95% confidence interval (CI): 1.01–9.38; *P*=0.049) and smaller ACD (OR, 0.05; 95% CI: 0.01–0.26; *P*=0.001) remained significant predictors for anterior capsule laxity.

**Table 3:** Predictors for anterior capsular laxity in eyes with angle closure.

Variable	Univariate Logistic Regression		Multivariate Logistic Regression			
	OR (95% CI)	*P* value	*β*	OR (95% CI)	*P* value	VIF
Age	1.03 (0.98–1.09)	0.257				
Sex, Female	0.89 (0.36–2.20)	0.807				
Diagnosis, APAC	5.16 (1.90–14.00)	0.001	1.110	3.04 (1.01–9.38)	0.049	1.741
Presence of VZ, yes	0.74 (0.29–1.88)	0.524				
CCT	1.00 (0.99–1.01)	0.609				
AK	0.96 (0.75–1.22)	0.721				
ACD	0.03 (0.00–0.31)	0.003	−2.900	0.05 (0.01–0.26)	0.001	1.123
LT	2.02 (0.68–6.04)	0.206				
AL	1.12 (0.68–1.84)	0.654				
AOD500, per 1 SD increase	0.55 (0.31–0.96)	0.037	−	−	−	1.573
TISA500, per 1 SD increase	0.63 (0.36–1.11)	0.112				
IT750, per 1 SD increase	0.83 (0.52–1.34)	0.451				
IC, per 1 SD increase	1.44 (0.93–2.21)	0.099	−	−	−	2.186
CBMT, per 1 SD increase	1.08 (0.65–1.79)	0.770				
ICPD, per 1 SD increase	1.89 (1.13–3.18)	0.016	−	−	−	2.441
CPA, per 1 SD increase	0.58 (0.34–1.01)	0.054	−	−	−	1.753

OR, odds ratio; CI, confidence interval; VIF, variance inflation factor; APAC, acute primary angle closure; VZ, vitreous zonule; CCT, central corneal thickness; AK, average keratometry; ACD, anterior chamber depth; LT, lens thickness; AL, axial length; AOD500, angle opening distance at 500 μm; TISA500, trabecular-iris space area at 500 μm; IT750, iris thickness at 750 μm from the iris insertion; IC, iris curvature; CMBT, ciliary body maximum thickness; ICPD, iris-ciliary process distance; CPA, ciliary process area; SD, standard deviation.

**Table 4:** Predictors for posterior capsular laxity in eyes with angle closure.

Variable	Univariate Logistic Regression		Multivariate Logistic Regression			
	OR (95% CI)	*P* value	*β*	OR (95% CI)	*P* value	VIF
Age	0.98 (0.94–1.02)	0.294				
Sex, Female	0.82 (0.37–1.86)	0.643				
Diagnosis, APAC	2.81 (1.06–7.45)	0.038	1.358	3.89 (1.20–13.50)	0.026	1.057
Presence of VZ, yes	0.22 (0.10–0.47)	<0.001	−2.247	0.11 (0.04–0.27)	<0.001	1.163
CCT	1.00 (0.99–1.01)	0.832				
AK	0.92 (0.74–1.14)	0.445				
ACD	0.39 (0.11–1.35)	0.137				
LT	2.41 (0.97–6.02)	0.059	–	–	–	1.025
AL	1.09 (0.71–1.68)	0.701				
AOD500, per 1 SD increase	0.91 (0.62–1.34)	0.637				
TISA500, per 1 SD increase	0.88 (0.60–1.31)	0.543				
IT750, per 1 SD increase	0.80 (0.55–1.16)	0.245				
IC, per 1 SD increase	1.34 (0.89–2.01)	0.163				
CBMT, per 1 SD increase	1.57 (1.04–2.37)	0.033	0.652	1.92 (1.22–3.17)	0.007	1.127
ICPD, per 1 SD increase	1.17 (0.80–1.72)	0.418				
CPA, per 1 SD increase	0.98 (0.66–1.44)	0.915				

OR, odds ratio; CI, confidence interval; VIF, variance inflation factor; APAC, acute primary angle closure; VZ, vitreous zonule; CCT, central corneal thickness; AK, average keratometry; ACD, anterior chamber depth; LT, lens thickness; AL, axial length; AOD500, angle opening distance at 500 μm; TISA500, trabecular-iris space area at 500 μm; IT750, iris thickness at 750 μm from the iris insertion; IC, iris curvature; CMBT, ciliary body maximum thickness; ICPD, iris-ciliary process distance; CPA, ciliary process area; SD, standard deviation.

For posterior capsule laxity, the univariable analysis identified diagnosis (APAC vs. PACD) (*P*=0.038), presence of VZ (*P*<0.001), LT (*P*=0.059) and CBMT (*P*=0.033) as potential predictors. In the multivariable model, the diagnosis of APAC (OR, 3.89; 95% CI: 1.20–13.50; *P*=0.026), presence of VZ (OR, 0.11; 95% CI: 0.04–0.27; *P*<0.001) and larger CBMT (OR, 1.92; 95% CI: 1.22–3.17; *P*=0.007) were significantly associated with posterior capsule laxity.

**Fig. 4:**
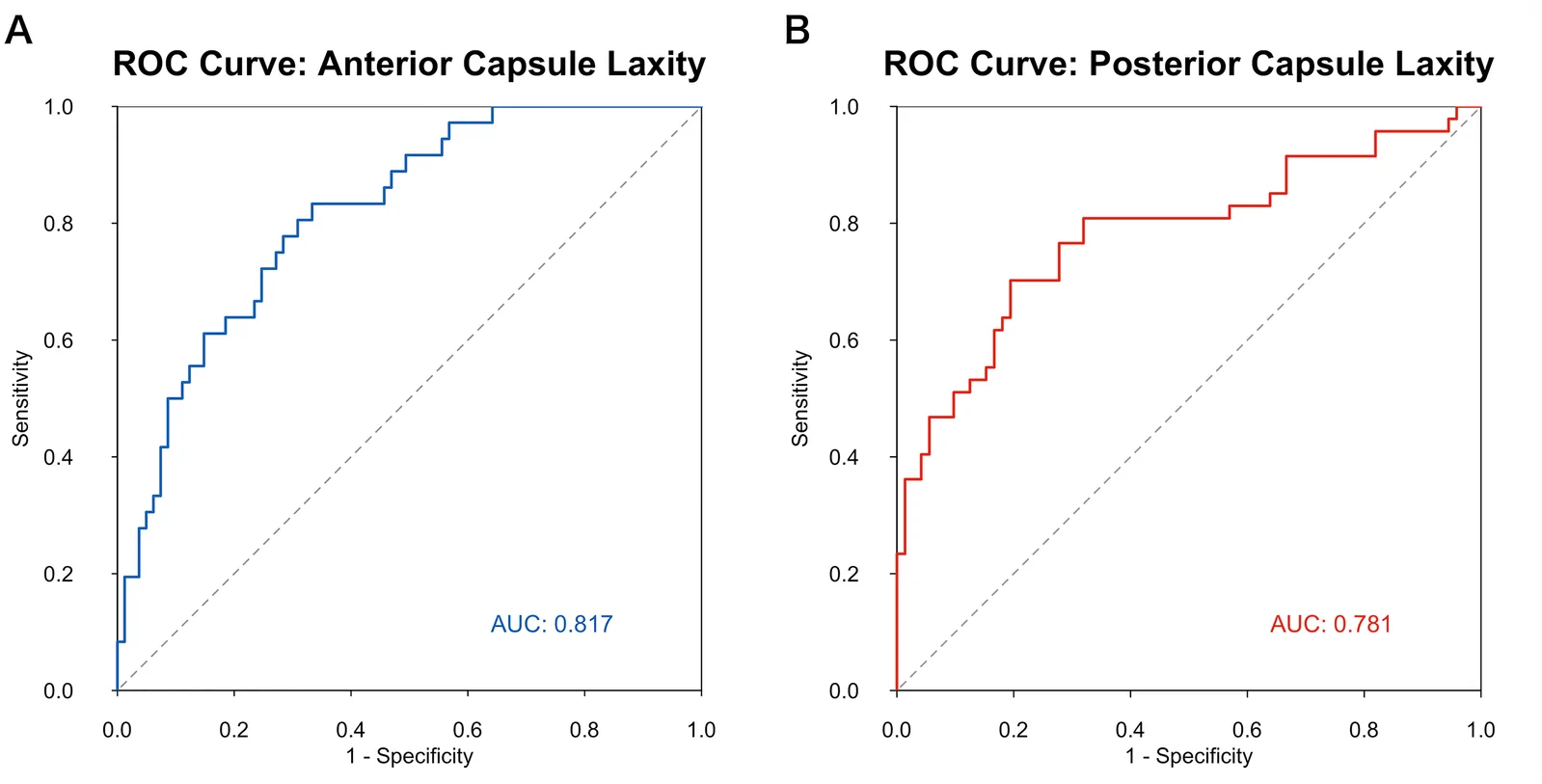
**Receiver Operating Characteristic Curves for Capsule Laxity Prediction Models.** (A) Anterior capsule laxity model (APAC diagnosis + ACD): AUC=0.817 (95% CI: 0.739–0.896). (B) Posterior capsule laxity model (APAC diagnosis + VZ absence + CBMT): AUC=0.781 (95% CI: 0.690–0.872). APAC, acute primary angle closure; ACD, anterior chamber depth; AUC, area under the curve; CI, confidence interval; VZ, vitreous zonule; CBMT, ciliary body maximum thickness.

The multivariable models yielded AUC values of 0.817 (95% CI: 0.739–0.896) for anterior capsule laxity and 0.781 (95% CI: 0.690–0.872) for posterior capsule laxity (Fig. 4). At the optimal cutoff determined by Youden index, the anterior capsule laxity model achieved a sensitivity of 83.3%, specificity of 66.7%, positive predictive value (PPV) of 52.6%, negative predictive value (NPV) of 90.0%, and accuracy of 71.8%. The posterior capsule laxity model demonstrated a sensitivity of 70.2%, specificity of 80.6%, PPV of 70.2%, NPV of 80.6%, and accuracy of 76.5% at an optimal predicted-probability cutoff of 0.427 (determined by the Youden index).

For clinical interpretability, we derived absolute cut-points using the Youden index from univariable ROC analyses. For anterior capsule laxity, the optimal univariable cut-off for ACD was ≤2.245 mm (Youden index), yielding sensitivity 75.0% and specificity 59.3% (univariable AUC for ACD=0.744). For CBMT (considered in relation to posterior capsule laxity), the univariable ROC yielded an optimal cutoff of ≥1.209 mm (AUC=0.618; sensitivity 36.2%, specificity 88.9%). This CBMT threshold is reported for transparency, but CBMT alone showed only modest discrimination.

In a sensitivity analysis restricted to PACD eyes (n=96, 23 with anterior capsule laxity, 73 with normal capsule), excluding all APAC cases, smaller ACD remained the sole independent predictor of anterior capsule laxity (adjusted OR, 0.05; 95% CI: 0.01–0.32; *P*=0.002), consistent with the primary analysis (Supplementary Table 3).

## 4 Discussion

Different intraoperative patterns of capsule laxity likely reflect zonular dysfunction at distinct anatomic locations and pose different surgical challenges that require tailored strategies. Eyes with anterior capsule laxity often need capsular hooks to complete the procedure safely, whereas eyes with posterior capsule laxity demand careful control of aspiration/flow during nucleus and cortical removal to reduce the risk of posterior capsule rupture.^10^^,^^25^^,^^26^ In cases of extensive anterior and/or posterior laxities, implantation of a capsular tension ring or other capsular support may be required to maintain long-term IOL-bag stability.^10^^,^^25^^,^^26^ However, at present, reliable preoperative assessment of capsular integrity remains limited, surgeons frequently confront unanticipated intraoperative challenges.^12^^,^^27^ Therefore, identifying preoperative imaging features that predict anterior or posterior capsule laxity would improve surgical planning, reduce complications, and optimize visual outcomes in patients with angle closure.

We observed that anterior capsule laxity was markedly more common among eyes presenting with APAC, and that eyes with anterior or combined capsule laxity had significantly shallower ACD. These results are consistent with prior reports showing high rates of intraoperative zonulopathy in APAC (reported range about 21%–69%) and with our earlier work that identified a critical ACD threshold (all APAC eyes with ACD ≤2.0 mm exhibited anterior capsule laxity).^8^^,^^10^^,^^12^^,^^14^

Furthermore, the regression analyses findings suggest that the diagnosis of APAC and shallower ACD independently predicted anterior capsule laxity. As reported in the Results, shallower ACD remained an independent predictor of anterior capsule laxity even after excluding APAC cases. Those findings suggest that the association between shallow ACD and anterior capsule laxity is not simply a byproduct of APAC, but rather reflects a consistent anatomical relationship within this angle-closure cohort. Mechanistically, this link may be bidirectional: preexisting zonulopathy may lead to lens thickening and anterior displacement, further shallowing the ACD and markedly increase APAC risk; conversely, APAC attacks and significantly elevated IOP may cause ischemia and inflammation in the ciliary body, thereby damaging the zonular fibers.^28^

Eyes with combined capsule laxity demonstrated greater LT than eyes with normal capsule. AS-OCT measurements in a representative subset confirmed greater LV in eyes with anterior and combined capsule laxity compared with normal capsule eyes, consistent with prior reports linking greater LT and LV to zonulopathy in PACD. A previous Chinese study identified LT >4.9 mm and LV >1.2 mm as risk thresholds.^9^^,^^10^^,^^29^ In eyes with combined capsule laxity, concurrent compromise of both CZs and VZs likely diminishes the direct and indirect posterior traction on the lens from the ciliary body, causing a more significant thickening of the lens with anterior lens displacement at the same time.^14^^,^^30^

Eyes with combined capsule laxity also showed increased ICPD together with reduced CPA, representing relative posterior–external shift and collapse of the ciliary apparatus. This combination likely reflects loss of the mechanical support normally transmitted by CZs.^14^ Once CZ integrity is compromised, the direct traction between the lens and ciliary processes is attenuated, permitting posterior and external displacement as well as structural remodeling of the ciliary processes.

We also found that both posterior and combined capsule laxity groups exhibited a markedly higher prevalence of VZ absence, compared to the normal-capsule group, and VZ absence was an independent predictor of posterior capsule laxity. Anatomically, VZs are the only zonular fibers that attach directly to and have direct force acting on the posterior capsule.^14^ Some previous studies reported reduced VZ visibility in angle closure eyes and associations between absent VZ and narrower angles.^18^^,^^31^, ^32^, ^33^

Complementing the VZ findings, CZ observations on quadrant UBM provided additional mechanistic insight. Although CZ strands were visible in most eyes, the number of quadrants with clearly identifiable CZ differed by capsular phenotype: eyes with combined laxity showed fewer quadrants with visible CZ compared with eyes with normal capsules. This reduced CZ quadrant count is compatible with more extensive CZ compromise in the combined-laxity phenotype and supports a model in which CZ disruption preferentially perturbs lens–ciliary coupling, whereas VZ loss preferentially undermines direct posterior-capsule support. Longitudinal and multimodal imaging studies are needed to clarify temporal and causal relationships among CZ integrity, VZ presence, and capsular laxity.

An increased CBMT on quadrant UBM in eyes with posterior capsule laxity should be interpreted with caution. Rather than implying cellular hypertrophy, the larger CBMT measurements are plausibly explained by positional or configurational changes of the ciliary processes (for example, relative anterior/medial displacement or altered cross-sectional geometry) that inflate thickness on quadrant scans. Loss of posterior tethering from the VZs could permit such reconfiguration, producing a larger apparent CBMT without true tissue proliferation.^14^^,^^23^

Apparent discrepancies between our CBMT findings and prior reports of thinner or unchanged ciliary bodies in APAC/PACG likely reflect differences in study populations (here, eyes selected by intraoperative capsule laxity vs. comparisons to fellow/healthy eyes), timing of imaging relative to acute IOP events, and measurement protocols (quadrant sampling versus central cross-section and landmark choice).^34^, ^35^, ^36^ Resolving these alternatives will require prospective, multimodal imaging (central cross-sectional AS-OCT, perfusion assessment) and longitudinal pre-/post-attack evaluation in well-phenotyped cohorts.

The clinical utility of the identified predictors (APAC diagnosis and shallower ACD for anterior capsule laxity; APAC diagnosis, absence of VZ and larger CBMT for posterior capsule laxity) was validated through ROC curve analysis, demonstrating good discriminative performance for predicting capsule laxity preoperatively. Since these biomarkers are derived from routine preoperative examinations for angle closure, surgeons can readily integrate this risk assessment into their clinical workflow to anticipate surgical challenges and optimize intraoperative management.

This study has several strengths. First of all, the relatively large sample size provides adequate statistical power for the regression analyses and enhances the reliability of our findings. Furthermore, to the best of our knowledge, this is the first study to identify specific predictors for anterior and posterior capsule laxity in Chinese patients with angle closure. Additionally, this study is among the first to systematically evaluate the role of VZs in capsule laxity, providing new insights into the mechanisms underlying posterior capsule instability.

Several limitations should be acknowledged. First, although we defined VZ as present if observed in at least one of four quadrants to reduce misclassification, VZ absence could still reflect imaging limitations rather than true anatomical loss. Second, this study included only Chinese patients, which may limit the external generalizability. Third, the outcome (capsule laxity) was defined by subjective intraoperative assessments, which could potentially be influenced by surgical technique variability. To minimize this bias, two senior surgeons recorded findings independently, surgeries were video-recorded, and discrepant cases were adjudicated by a masked senior reviewer, but residual subjectivity remains. Fourth, because operating surgeons had access to preoperative UBM, confirmation bias cannot be fully excluded despite the above safeguards. Finally, some patients contributed both eyes; we used correlation-adjusted models to account for inter-eye dependence, but inclusion of bilateral eyes may still introduce residual bias.

## 5 Conclusions

Our study identified the diagnosis of APAC and shallower ACD as potential biomarkers for anterior capsule laxity, and VZ absence, the diagnosis of APAC along with larger CBMT as biomarkers for posterior capsule laxity in Chinese patients with angle closure. Awareness of these preoperative predictors may assist surgeons in anticipating surgical challenges and optimize intraoperative management.

## Study approval

The authors confirm that any aspect of the work covered in this manuscript that involved human patients or animals was conducted with the ethical approval of all relevant bodies and the study was performed in accordance with the Declaration of Helsinki，and the protocol was approved by the Ethics Committee of Beijing Tongren Hospital, Capital Medical University (Approval No. TREC2022-KY003).

## Author contributions

The authors confirm contribution to the paper as follows: Data curation, Conceptualization, Methodology, Writing original draft: HZ; Conceptualization, Formal analysis, Methodology, Writing original draft: JW; Data curation, Investigation, Resources: YW, DL, XT; Data curation, Methodology, Investigation: ZL; Methodology, Software, Formal analysis: LS; Methodology, Software, Resources: CQ; Methodology, Writing – review and editing: CC; Conceptualization, Data curation, Methodology, Investigation, Project administration, Supervision, Writing – review and editing: YZ. All authors reviewed the results and approved the final version of the manuscript.

## Funding

This research did not receive any specific grant from funding agencies in the public, commercial, or not-for-profit sectors.

## Declaration of competing interest

The authors declare that they have no known competing financial interests or personal relationships that could have appeared to influence the work reported in this paper.
