# Supplementary material for: Anterior chamber depth and vitreous zonule integrity as biomarkers for intraoperative capsule laxity in angle closure
Source: Adv Ophthalmol Pract Res. 2026 May 1;6(3):257–65. doi: 10.1016/j.aopr.2026.04.002 (PMC13495364; doi:10.1016/j.aopr.2026.04.002)
Supplement: Supplementary file 1 [file mmc1.docx]

**Supplementary Table 1. Demographic Characteristics of the Included Patients with Angle Closure**

| **Parameter** | **Median (IQR) / Number (%)** |
| --- | --- |
| Participants | 203 |
| Age, years | 68.0 (62.0, 72.0) |
| Sex |  |
| Male  Female | 65 (32.0)  138 (68.0) |
| Diagnosis |  |
| PAC  PACG  APAC | 43 (21.2)  111 (54.7)  49 (24.1) |
| Family history of angle closure |  |
| No  Yes | 185 (91.1)  18 (8.9) |
| Hypertension |  |
| No  Yes | 136 (67.0)  67 (33.0) |
| Diabetes |  |
| No  Yes | 171 (84.2)  32 (15.8) |
| Hyperlipidemia |  |
| No  Yes | 175 (86.2)  28 (13.8) |

IQR, interquartile range; PAC, primary angle closure; PACG, primary angle closure glaucoma; APAC, acute primary angle closure.
