# Supplementary material for: Anterior chamber depth and vitreous zonule integrity as biomarkers for intraoperative capsule laxity in angle closure
Source: Adv Ophthalmol Pract Res. 2026 May 1;6(3):257–65. doi: 10.1016/j.aopr.2026.04.002 (PMC13495364; doi:10.1016/j.aopr.2026.04.002)
Supplement: Supplementary file 2 [file mmc2.docx]

**Supplementary Table 2. ASOCT Parameters in Included Angle Closure Eyes with Different Capsular Conditions**

| **Parameter** | **1 = Anterior capsule laxity group (*n* = 14)** | **2 = Posterior capsule laxity group (*n* = 17)** | **3 = Combined capsule laxity group (n = 22)** | **4 = Normal capsule group**  **(n = 16)** | ***P* value** | ***P* value (1 &4)** | ***P* value (2 &4)** | ***P* value (3 & 4)** | ***P* value (1 & 2)** |
| --- | --- | --- | --- | --- | --- | --- | --- | --- | --- |
| ACW (SD), mm | 11.126 ± 0.309 | 11.018 ± 0.379 | 11.298 ± 0.446 | 11.084 ± 0.526 | 0.207* |  |  |  |  |
| ACA (SD), mm^2^ | 10.710 ± 2.227 | 11.663 ± 1.982 | 11.910 ± 1.793 | 12.806 ± 2.318 | 0.058* |  |  |  |  |
| IA (SD), mm^2^ | 2.687 ± 0.474 | 2.617 ± 0.327 | 2.819 ± 0.365 | 2.607 ± 0.566 | 0.442* |  |  |  |  |
| LV (SD), mm | 1.300 ± 0.376 | 1.033 ± 0.245 | 1.253 ± 0.320 | 0.907 ± 0.338 | 0.002* | 0.008§ | 1.000§ | 0.010§ | 0.144§ |
| R (Front) (SD), mm | 7.44 ± 0.79 | 8.08 ± 1.07 | 7.92 ± 0.60 | 8.21 ± 0.83 | 0.087* |  |  |  |  |
| R (Back) (SD), mm | 5.76 ± 0.62 | 5.34 ± 0.55 | 5.64 ± 0.74 | 5.45 ± 0.72 | 0.327* |  |  |  |  |
| Lens decentration (SD), mm | 0.07 ± 0.19 | 0.02 ± 0.16 | -0.00 ± 0.11 | 0.03 ± 0.11 | 0.486* |  |  |  |  |
| Lens equator diameter (SD), mm | 10.16 ± 0.64 | 9.89 ± 0.73 | 10.08 ± 0.97 | 9.85 ± 0.83 | 0.703* |  |  |  |  |
| PD (IQR), mm | 4.91 (4.20, 5.25) | 4.09 (3.50, 5.13) | 4.62 (3.72, 5.12) | 4.50 (4.16, 5.52) | 0.469† |  |  |  |  |

ASOCT, anterior segment optical coherence tomography; ACW, anterior chamber width; ACA, anterior chamber area; IA, iris cross-sectional area; LV, lens vault; R (Front), the radius of the front curvature of lens; R (Back), the radius of the back curvature of lens; PD, pupil diameter; SD, standard deviation; IQR, interquartile range.

† Kruskal-Wallis test. ‖ Mann–Whitney test (<0.05/6 = 0.008 = significant difference). * The 1-way ANOVA. § Bonferroni test.
