# Supplementary material for: Anterior chamber depth and vitreous zonule integrity as biomarkers for intraoperative capsule laxity in angle closure
Source: Adv Ophthalmol Pract Res. 2026 May 1;6(3):257–65. doi: 10.1016/j.aopr.2026.04.002 (PMC13495364; doi:10.1016/j.aopr.2026.04.002)
Supplement: Supplementary file 3 [file mmc3.docx]

**Supplementary Table 3.** Sensitivity Analysis: Predictors for Anterior Capsule Laxity in PACD Eyes (Excluding APAC)

| **Variable** | **Univariate Analysis** | | **Multivariate Analysis** | | |
| --- | --- | --- | --- | --- | --- |
|  | **OR (95% CI)** | **P value** | **OR (95% CI)** | **P value** | **VIF** |
| Age | 1.04 (0.99, 1.10) | 0.124 |  |  |  |
| Sex, Female | 0.98 (0.35, 2.75) | 0.967 |  |  |  |
| Presence of VZ, yes | 0.91 (0.28, 2.93) | 0.878 |  |  |  |
| AK | 0.90 (0.67, 1.22) | 0.487 |  |  |  |
| ACD | 0.03 (0.01, 0.15) | <0.001 | 0.05 (0.01, 0.32) | 0.002 | 1.026 |
| LT | 0.82 (0.54, 6.58) | 0.133 |  |  |  |
| AL | 1.11 (0.58, 2.11) | 0.759 |  |  |  |
| AOD500, per 1 SD increase | 0.99 (0.99, 1.00) | 0.099 | -- | -- | 1.275 |
| TISA500, per 1 SD increase | 1.00 (1.00, 1.00) | 0.148 |  |  |  |
| IT750, per 1 SD increase | 1.00 (0.99, 1.01) | 0.850 |  |  |  |
| IC, per 1 SD increase | 1.00 (1.00, 1.01) | 0.184 |  |  |  |
| CBMT, per 1 SD increase | 1.00 (1.00, 1.01) | 0.771 |  |  |  |
| ICPD, per 1 SD increase | 1.00 (1.00, 1.01) | 0.050 | -- | -- | 1.930 |
| CPA, per 1 SD increase | 1.00 (1.00, 1.00) | 0.029 | -- | -- | 1.602 |
|  |  |  |  |  |  |

PACD, primary angle closure disease; APAC, acute primary angle closure; OR, odds ratio; CI, confidence interval; VIF, variance inflation factor; VZ, vitreous zonule; AK, average keratometry; ACD, anterior chamber depth; LT, lens thickness; AL, axial length; AOD500, angle opening distance at 500 μm; TISA500, trabecular-iris space area at 500 μm; IT750, iris thickness at 750μm from the iris insertion; IC, iris curvature; CBMT, ciliary body maximum thickness; ICPD, iris-ciliary process distance; CPA, ciliary process area; SD, standard deviation.

*Generalized estimating equations (GEE) with exchangeable correlation structure were used to account for inter-eye correlation.*
